# Lifestyle behaviours do not moderate the association between childhood maltreatment and comorbid depression and cardiometabolic disease in older adults: a meta-analysis

**DOI:** 10.1186/s12916-025-03950-1

**Published:** 2025-03-05

**Authors:** Olujolagbe Layinka, Camille Souama, Serena Defina, Vilte Baltramonaityte, Charlotte A. M. Cecil, Punit Shah, Yuri Milaneschi, Femke Lamers, Brenda W. J. H. Penninx, Esther Walton

**Affiliations:** 1 Department of Psychology, University of Bath, Building 10 West, Bath, BA2 7AY UK; 2 Department of Psychiatry, Amsterdam UMC Vrije Universiteit Amsterdam, De Boelelaan 1117, Amsterdam, The Netherlands; 3 Amsterdam Public Health, Mental Health Program, Amsterdam, The Netherlands; 4 Amsterdam Neuroscience, Mood, Anxiety, Psychosis, Stress, and Sleep Program, Amsterdam, The Netherlands; 5 Department of Child and Adolescent Psychiatry, Erasmus MC, University Medical Center Rotterdam, Rotterdam, The Netherlands; 6 Generation R Study Group, Erasmus MC, University Medical Center Rotterdam, Rotterdam, The Netherlands; 7 Department of Child and Adolescent Psychiatry, Erasmus MC, University Medical Center Rotterdam, Rotterdam, The Netherlands; 8 Department of Epidemiology, Erasmus MC, University Medical Center Rotterdam, Rotterdam, The Netherlands; 9 Department of Biomedical Data Sciences, Molecular Epidemiology, Leiden University Medical Center, Leiden, The Netherlands

**Keywords:** Physical activity, Smoking, Alcohol, Childhood maltreatment, Depression, Cardiometabolic disease, Comorbidity, Meta-analysis

## Abstract

**Background:**

Comorbidity between depression and cardiometabolic diseases is an emerging health concern, with childhood maltreatment as a major risk factor. These conditions are also linked to unhealthy lifestyle behaviours such as physical inactivity, smoking, and alcohol intake. However, the precise degree to which lifestyle behaviours moderate the risk between childhood maltreatment and comorbid depression and cardiometabolic disease is entirely unknown.

**Methods:**

We analysed clinical and self-reported data from four longitudinal studies (N_pooled_ = 181,423; mean follow-up period of 5–18 years) to investigate the moderating effects of physical activity, smoking, and alcohol intake, on the association between retrospectively reported childhood maltreatment and i) depression, ii) cardiometabolic disease and iii) their comorbidity in older adults (mean age range of 47–66 years). Estimates of these moderation effects were derived using multinomial logistic regressions and then meta-analysed.

**Results:**

No meaningful moderation effects were detected for any of the lifestyle behaviours on the association between childhood maltreatment and each health outcome. Physical activity was linked to lower odds of depression (OR [95% CI] = 0.94 [0.92; 0.96]), while smoking was a risk factor for all three outcomes (OR [95% CI] = 1.16 [1.04; 1.31] or larger). Alcohol intake was associated with slightly lower odds of comorbidity (OR [95% CI] = 0.69 [0.66; 0.73]), although this association was not stable across all sensitivity analyses.

**Conclusions:**

Lifestyle behaviours did not moderate the risk association between childhood maltreatment and depression, cardiometabolic disease, and their comorbidity in older adults. However, we confirmed that childhood maltreatment was associated with these conditions. Further research should address the limitations of this study to elucidate the most optimal targets for intervention.

**Supplementary Information:**

The online version contains supplementary material available at 10.1186/s12916-025-03950-1.

## Background

Depression and cardiometabolic diseases such as diabetes and ischemic heart disease consistently represent the leading global causes of adult disability [1]. These conditions are strongly comorbid and bidirectionally associated [2–4]. Individuals with depression are up to 65% more likely to develop a cardiometabolic disease compared to individuals without depression [5, 6], and 17–44% of individuals with coronary artery disease are also diagnosed with major depression [7, 8]. This bidirectional relationship likely stems from shared genetic and environmental vulnerabilities, leading to causally associated biological responses and behaviours underlying each condition [3, 8–12]. The resulting elevated morbidity and mortality rates pose a major public health issue worldwide.

A growing body of research, including our previous meta-analysis by Souama et al. [8], indicates that childhood maltreatment is a critical factor that can amplify the risk of developing comorbid depression and cardiometabolic disease in adulthood, more so than each of these conditions in isolation [8, 13]. Notably, the cumulative impact of childhood maltreatment follows a dose–response relationship, where the amalgamation of stressors increases the likelihood of developing at least one health condition [13]. Thus, the more maltreatment experienced, the higher the risk of depression and cardiometabolic diseases, as well as their co-occurrence [14, 15]. Further, this risk association may emerge as early as childhood, before the onset of disease. Defina et al. [16] observed that early-life stress experienced in childhood was linked to both higher internalising symptoms and increased fat mass in adolescents, preclinical markers for depression and cardiometabolic diseases. These findings support the notion that chronic stress induced by childhood maltreatment may contribute to the development of depression and cardiometabolic comorbidity [17].

Lifestyle behaviours such as smoking, excessive alcohol intake, and a lack of regular physical activity have been linked to chronic stress and an increased likelihood of depression and cardiometabolic diseases [18–22]. Conversely, modest increases to physical activity can weaken the cumulative physical and psychological burden of chronic stress [23], and are associated with a reduced risk of depression [24]. Childhood maltreatment is also associated with an increased propensity for engaging in these behaviours [25]. As these lifestyle behaviours are modifiable, they present as promising targets for the prevention of depression and cardiometabolic diseases, particularly in people with a history of maltreatment. However, the extent and developmental period at which the modification of these lifestyle behaviours can affect the association between childhood maltreatment and the risk of comorbid depression and cardiometabolic disease is unknown. Our previous study by Defina et al. [16] found that engaging in healthy lifestyle behaviours did not have a mitigating effect on the association between childhood maltreatment and this comorbidity among adolescents. However, it is possible that moderating effects only emerge later in life. Understanding the emergence of these effects across the lifespan is paramount for the development of interventions that may help mitigate the effects of childhood maltreatment on adult comorbidity.

In the present follow-up study, we advanced previous investigations conducted by Souama et al. [8] and Defina et al. [16] by exploring the moderating role of lifestyle behaviours in the relationship between childhood maltreatment and depression, cardiometabolic disease, and their comorbidity in adult populations. We aim to elucidate whether such moderating associations emerge in late adulthood. As such, our research aimed to enhance our understanding regarding the role of lifestyle behaviours as potential modifiers in the intricate relationship between childhood maltreatment and the risk of comorbid depression and cardiometabolic diseases in adulthood.

## Methods

### Studies and participants

Following our previous studies by Souama et al. [8] and Defina et al. [16], we included longitudinal studies that are part of the EarlyCause consortium [26] with relevant data on childhood maltreatment, depression, cardiometabolic health and lifestyle behaviours (smoking, alcohol intake, and physical activity). To minimize the potential for reverse causation (i.e., a disease diagnosis leading to an engagement in more beneficial lifestyle behaviours), we included only studies that measured lifestyle behaviours before disease. In total, data from four studies from the UK and the Netherlands were included; UK Biobank [27, 28], Netherlands Study of Depression and Anxiety (NESDA) [29, 30], and Avon Longitudinal Study of Parents And Children (ALSPAC) mothers and partners [31, 32] (Additional file 1: Fig. S1, Table S1, and Sect. 1) [8, 29, 31–35]. Three studies were population-based while one was a case–control cohort with an overrepresentation of affective disorders (N_pooled_ = 181,423).

All participants provided informed consent and ethical approval for each study was obtained from local research ethics committees (see Additional file 1: Sect. 1.1) [29, 31–35].

## Measures

### Exposures

#### Childhood maltreatment

Retrospective self-reported childhood maltreatment was measured using the Childhood Trauma Screener [36] in UK Biobank, the Childhood Trauma Interview [37] in NESDA, and a single item question in ALSPAC mothers and partners (see Additional file 1: Table S1). The presence of childhood maltreatment was assessed in any of the following categories: physical, emotional, and/or sexual abuse, before the age of 18. Retrospective self-reported measures were completed at baseline (mean age: 57 years (y) in UK Biobank, 42y in NESDA, 29y in ALSPAC mothers, and 30y in ALSPAC partners). Physical and emotional maltreatment were defined by: (1) self-reported history of frequent abuse (e.g., “often”, “very often”, “regularly”) when a frequency assessment was available (UK Biobank, NESDA) or (2) self-reported history of abuse in cases of a dichotomous assessment (ALSPAC mothers and partners). Sexual abuse was defined by the report of at least one occurrence of sexual abuse in childhood. Specific measures and criteria used to code childhood maltreatment (absent vs. present) in each study are described in Additional file 1: Table S1 and Souama et al. [8].

### Outcomes

Only participants who developed depression and cardiometabolic disease after baseline were included in this study to minimize reverse causation (average ages at the first follow up were 61y in UK Biobank, 44y in NESDA, 48y in ALSPAC mothers, and 53y in ALSPAC partners; Additional file 1: Table S2-3). We note that the median age on onset for depression is around age 30 [38] and age 45 for type 2 diabetes [39]. Thus, we specify throughout that our findings are related to older adults.

#### Depression

Depression was assessed at the most recent time point(s) available in each cohort (6-11y follow-ups/61-66y in UK Biobank, 2-6y follow-ups/44-48y in NESDA, 18y follow-up/48y in ALSPAC mothers, and 21-22y follow-up/53y in ALSPAC partners). Depression was defined as: (1) the presence of a lifetime diagnosis of major depressive disorder assessed with (semi-)structured clinical interviews or (2) current depressive symptomatology assessed with self-report scales using validated clinical cut-offs. Specifically, depression was measured using a two-item questionnaire in UK Biobank, the Composite International Diagnostic Interview [40] in NESDA, and the Edinburgh Postnatal Depression Scale [41] in ALSPAC mothers and partners (see Additional file 1: Table S2). To account for pre-existing depression (already present at the time when lifestyle behaviours were assessed), individuals who had depression before the first reports of lifestyle behaviours (at baseline in UK Biobank and NESDA and up to 2y follow-up in ALSPAC mothers and partners) were excluded. Cohort-specific measures and criteria used to identify depression cases (absent vs. present) are described in Additional file 1: Table S2 and Souama et al. [8].

#### Cardiometabolic disease

Cardiometabolic disease was measured using dichotomous three to four item questionnaires in UK Biobank, NESDA, and ALSPAC mothers and partners (see Additional file 1: Table S3). Cardiometabolic disease was assessed at the same time points as depression (6-11y follow-up in UK Biobank, 2-6y follow-up in NESDA, 18y follow- up in ALSPAC mothers, and 21-22y follow-up in ALSPAC partners) and defined based on self-reports of a lifetime clinical diagnosis of diabetes mellitus (absent vs. present) and/or cardiovascular disease, where available (stroke, heart attack, angina or blood clots). In cohorts where this information was available (UK Biobank and NESDA), individuals who had lifetime or current cardiometabolic disease before the first reports of lifestyle behaviours were excluded. This was to account for pre-existing cardiometabolic disease (already present when lifestyle behaviours were assessed). Cohort-specific measures and criteria used to identify cardiometabolic disease cases (absent vs. present) are described in Additional file 1: Table S3 and Souma et al. [8].

#### Comorbidity

Aligned with our previous study [8], comorbidity was defined as a factor with levels as follows: 0 = absence of depression and cardiometabolic disease (healthy controls), 1 = depression only, 2 = cardiometabolic disease only, 3 = comorbidity of depression and cardiometabolic disease.

### Moderating lifestyle behaviours

#### Smoking

Current smoking was measured using a single self-reported question that captured the number of cigarettes smoked per day (Additional file 1: Table S4). In UK Biobank and NESDA, current smoking was assessed at baseline. In ALSPAC mothers and partners, this was assessed and averaged across four follow-up time points (21, 33, 61 and 85 month (m) follow-ups in mothers; 8, 21, 33, and 61m follow-ups in partners).

#### Alcohol intake

Current alcohol intake was measured using a single question. In UK Biobank, this was self-reported at baseline as: “never”, “special occasions only”, “1-3 times a week”, “3-5 times a week”, or “daily or almost daily”. In NESDA, current alcohol intake was self-reported at baseline as: “never”, “monthly or less”, “2-4 times per month”, “2-3 times per week”, or “+4 times per week”. In ALSPAC this was self-reported in mothers (averaged across 21, 33 and 61m follow-ups) and partners (averaged across 21, 33, 47 and 61m follow-ups) as: “never”, “less than once a week”, “at least once a week”, “1-2 glasses nearly every day”, “3-9 glasses every day”, or “at least 10 glasses a day”. See Additional file 1: Table S5 for details.

#### Physical activity

Physical activity was measured using three items in UK Biobank, six items in NESDA, several items in ALSPAC mothers, and eight items in ALSPAC partners. Physical activity was classed as: current number of hours per week spent doing light, moderate, and vigorous physical activity in UK Biobank and NESDA, a combination of both frequency (time per week) and duration (hours per week) in ALSPAC mothers; or frequency of physical activity in the past week/year in ALSPAC partners. This was self-reported: at baseline in UK Biobank and NESDA, averaged across three follow-up time points (7y, 8y and 11y follow-ups) in ALSPAC mothers, and at 7y follow-up in ALSPAC partners. Activities in ALSPAC included sports such as swimming, running, cycling, tennis, badminton, aerobics, netball, and more. See Additional file 1: Table S6 for details.

### Covariates

As covariates, we included sex, ethnicity, and educational attainment (assessed at the earliest available timepoint) and age at outcome (i.e., depression, cardiometabolic disease and their co-occurrence; assessed at the most recent time point). Ethnicity was coded as White / Non-White (or North-European / Non-North-European in NESDA). As reported in Souama et al. [8], educational attainment was based exclusively on self-reports, harmonized across cohorts and countries by using the International Standard Classification of Education (ISCED) 2011, [42] and categorized in three levels: ISCED 0–2 corresponds to no education, early childhood education, primary and lower secondary education; ISCED 3–4 corresponds to upper secondary education and postsecondary non-tertiary education; and ISCED 5–8 corresponds to short-cycle tertiary education, bachelor, master, and doctor or equivalent levels.

### Statistical analysis

We ran analyses separately in all four cohorts and then meta-analysed results across datasets. On average, 9.8% of data was missing in UK Biobank, 3.8% in NESDA, 8.9% in ALSPAC mothers, and 12.0% in ALSPAC partners. Missing data (i.e., exposures, outcomes, moderators and covariates) were imputed using multiple imputation (40 iterations over 20 imputations), as implemented in the ‘mice’ R package [43]. In detail, continuous measures were imputed using random forest, binary variables using logistic regression, and unordered categorical data with more than two levels using polytomous regression. Model parameters were fit in each imputed dataset and then pooled according to Rubin’s Rules.

In the main analyses, the associations of childhood maltreatment, lifestyle behaviours, and their interaction (i.e., lifestyle behaviour moderating effect) on depression, cardiometabolic disease and comorbidity were assessed using multinomial logistic regression. The reference level was set to “unaffected controls”, meaning no depression or cardiometabolic diseases. All models were corrected for sex, ethnicity, educational attainment and age at outcome. Separate models were used for each lifestyle behaviour. Moderators were standardized to generate estimates that could be easily compared across models. Heterogeneity was assessed using the I^2^ statistic [44], where >50% indicates substantial to considerable heterogeneity [45].

### Sensitivity analyses

In addition to assessing the moderating effect of lifestyle behaviours that were measured continuously, measures were binarized using – where available – recommended guidelines [46–48]. The continuous measure of smoking (‘number of cigarettes smoker per day’) was dichotomized into *smokers* and *non-smokers/ former smokers* (at baseline). Alcohol intake was dichotomized into *problematic* (daily or almost daily in UK Biobank; > 3 times per week in NESDA; > 2 glasses every day in ALSPAC) vs *non-problematic* drinking. Physical activity was dichotomized into *recommended* (at least 150 minutes of walking or moderate activity per week or 75 minutes of vigorous activity in UK Biobank; >2 times per week of vigorous activity of at least 20 minutes per day or >4 days per week of moderate activity or walking of at least 30 minutes per day or a minimum of 600 MET (metabolic equivalent total) minutes per week in NESDA; >3 times per week in ALSPAC) vs *insufficient*. Analyses were also repeated 1) excluding UK Biobank as the largest contributing cohort, 2) on unimputed (original) data and 3) without the interaction effect (for main effects only). We used the statistical software R and the ‘metafor’ package [49] for analysis. Scripts used for analyses are publicly available at https://github.com/stegosaurusrox/childhood.maltr_lifestyle_multimorb_adults.

## Results

### Sample descriptives

We included data across four longitudinal cohorts, with a total sample size of N_pooled_ = 181,423 (Table 1). The proportion of females in the two sex-mixed cohorts were 56% (UK Biobank) and 64% (NESDA). Mean age at baseline ranged from 29 to 56 years, with an average follow-up period between 5 to 18 years. Participants in all cohorts were mostly White or of North-European descent (all > 95%), but educational levels were varied across cohorts (proportion with a university degree ranged from 15 to 57%). Nine to 20% of participants reported a history of childhood maltreatment. During the follow-up period, 5 to 18% developed depression, while 4 to 10% reported a new diagnosis of cardiometabolic disease. Comorbidity developed after baseline was reported in 1 to 2% of individuals per cohort.

**Table 1:** Descriptive statistics of all cohorts

Cohort	UK Biobank	NESDA	ALSPAC mothers	ALSPAC partners
*N*	165747	904	9537	5235
Age in years at baseline (Mean, Range)	56 (38–72)	41 (18–65)	29 (15–44)	31 (16–65)
Age in years at most recent follow-up (Mean, Range)	66 (50–82)	46 (21–72)	48 (34–63)	53 (38–82)
Sex (*n*, % Female)	92700 (56)	582 (64)	9537 (100)	5235 (0)
Ethnicity (*n*, % White or North-European descent)	160861 (97)	866 (96)	9349 (98)	5151 (98)
Education (*n*, % University Degree)	94735 (57)	149 (16)	1418 (15)	1385 (26)
Childhood maltreatment (*n*, % Yes)	18996 (12)	175 (20)	1059 (11)	466 (9)
Comorbidity				
Unaffected (*n*, % Yes)	134474 (81)	694 (77)	5308 (56)	4059 (76)
Depression at most recent follow-up (*n*, % Yes)	17145 (10)	163 (18)	728 (8)	272 (5)
Cardiometabolic disease at most recent follow up (*n*, % Yes)	11095 (7)	32 (4)	761 (8)	499 (10)
Comorbidity at most recent follow-up (*n*, % Yes)	1623 (1)	15 (2)	135 (1)	38 (1)
Alcohol intake (Mean, Range)^a^	2 (0–4)	3 (1–5)	2 (1–6)	3 (1–9)
Problematic alcohol intake (*n*, % Yes)	38511 (23)	241 (27)	85 (1)	353 (7)
Physical activity frequency^b^ or hours per week^c^ (Mean, Range)	8 (0–63)^c^	14 (0–63)^c^	51 (1–217)^d^	4 (1–6)^b^
Recommended physical activity (*n*, % Yes)	135399 (82)	715 (79)	1500 (16)	1606 (31)
Cigarettes per day (Mean, Range)	1 (0–30)	7 (0–72)	3 (0–30)	3 (0–30)
Smoker (*n*, % Yes)	11765 (7)	270 (30)	2705 (28)	1595 (31)

^a^For alcohol level coding, see Additional file 1: Table S5. Physical activity was measured in ^b^frequency (times per week) or ^c^duration (hours per week). ^d^In ALSPAC mothers, mixed reports of frequency and durations were rank-normalized across different scales and time points, therefore, numeric values have no meaningful real-life unit. For level coding, see Additional file 1: Table S6

**Fig. 1:**
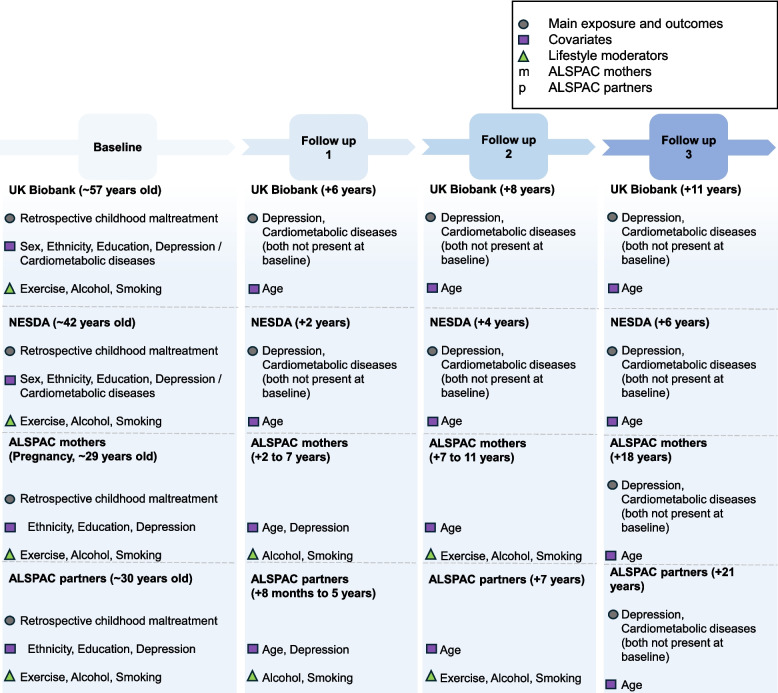
Overview of studies, measures, and time points included in the current study. Main exposure and outcomes (circles), covariates (squares), and lifestyle moderators (triangles) are highlighted. ALSPAC was split into two separate sub-studies of mothers and partners, resulting in a total of four studies (UK Biobank, NESDA, ALSPAC mothers and partners)

Alcohol intake varied across cohorts, with the most commonly reported frequency being ‘once or twice a week’ (UK Biobank), ‘2-4 times a month’ (NESDA), ‘less than once a week’ (ALSPAC mothers), and ‘at least once a week’ (ALSPAC partners). Problematic alcohol intake ranged from 1 to 27%. Seven to 31% were smokers, and participants smoked on average 1 to 7 cigarettes per day. On average, individuals were physically active for 8 (UK Biobank) and 14 hours per week (NESDA), or 1-3 times per month (ALSPAC partners), with 16 to 82% of participants achieving the recommended levels for physical activity (Fig. 1).

### Main analysis

**Fig. 2:**
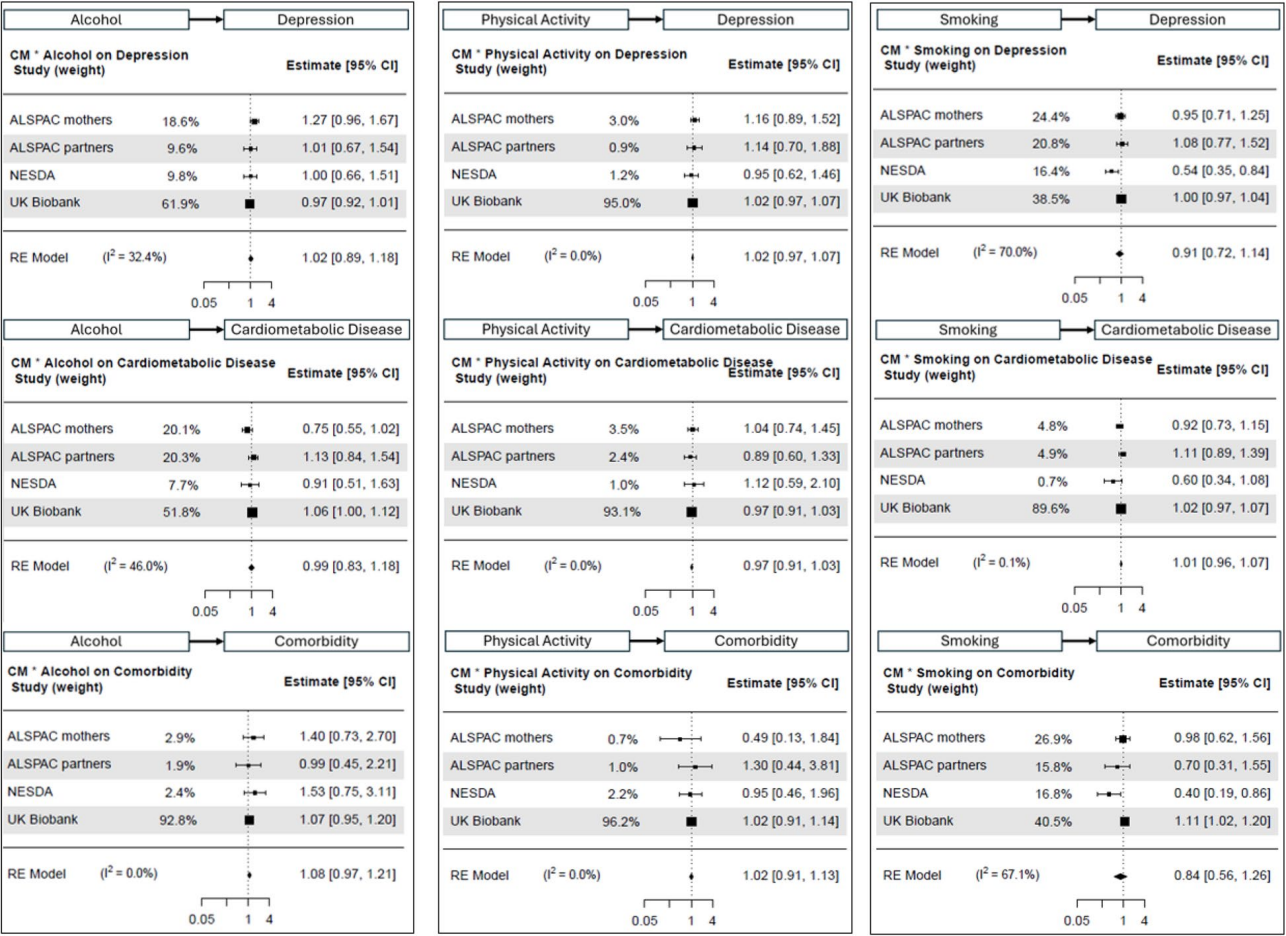
Childhood maltreatment by lifestyle behaviour interaction effect on depression (top row), cardiometabolic disease (middle row) and comorbidity (bottom row). Alcohol model shown in the left column, physical activity in the middle column, and smoking in the right column. Lifestyle behaviours were modelled continuously

Interaction effects detected between childhood maltreatment and each of the three lifestyle behaviours, on each of the three outcomes, were very weak and not meaningful (ORs between 0.84 to 1.08; I^2^ between 0 to 70%; Fig. 2).

Childhood maltreatment was associated with meaningfully higher odds of depression (ORs [95% CI] = 1.58 [1.27; 1.96] to 1.61 [1.29; 2.01; I^2^ between 64 to 66%] across the three lifestyle models), cardiometabolic disease (OR [95% CI] = 1.31 [1.08; 1.59] to 1.42 [1.10; 1.83] ; I^2^ between 55 to 79%) and their comorbidity (OR [95% CI] = 1.95 [1.36; 2.79] to 2.00 [1.44; 2.78]; I^2^ between 42 to 47%; Additional file 1: Fig. S1) in older adults.

**Fig. 3:**
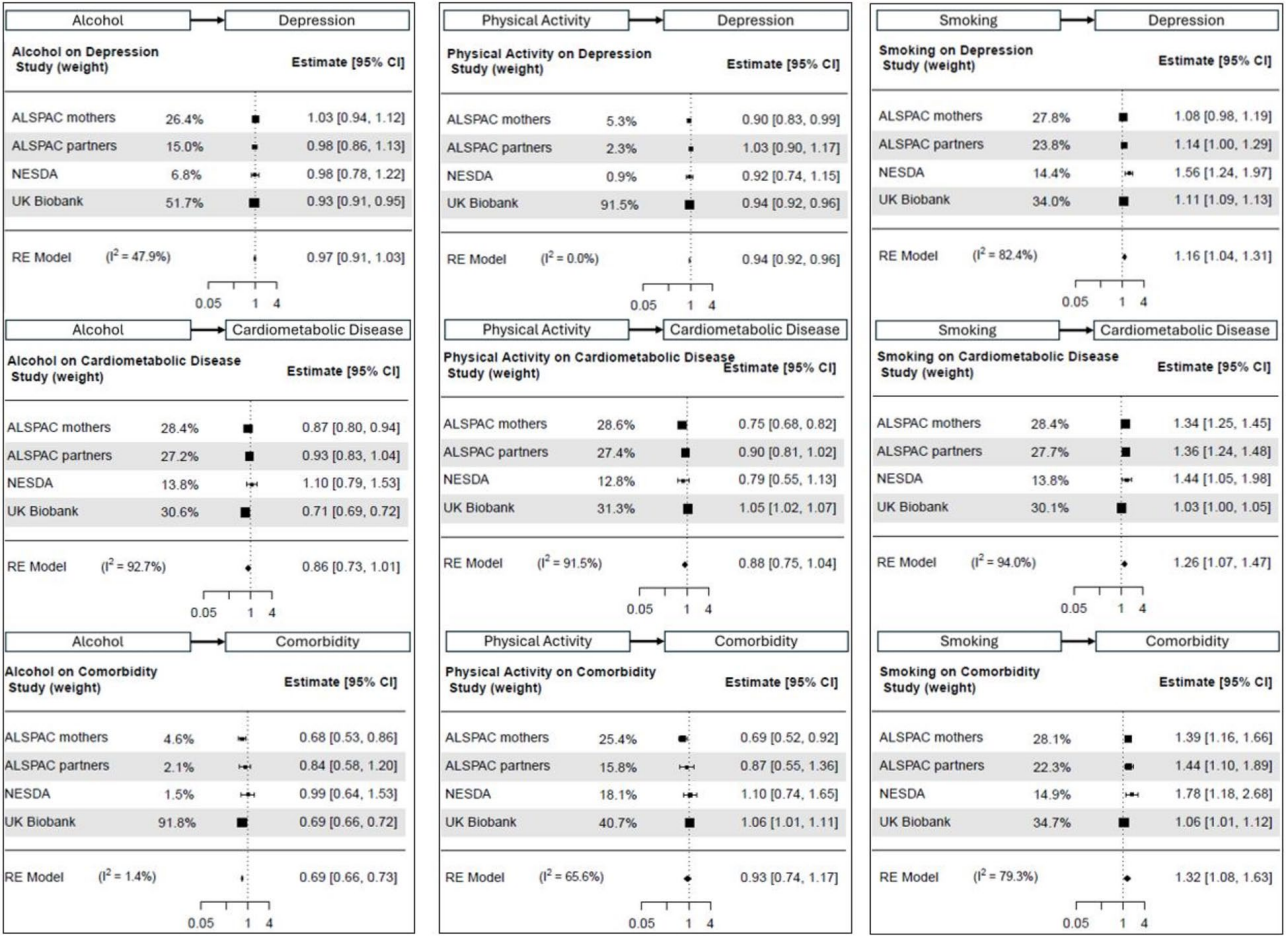
Main effect of alcohol intake (left column), physical activity (middle column) and smoking (right column) on depression (top row), cardiometabolic disease (middle row) and comorbidity (bottom row)

Perhaps surprisingly, alcohol intake was associated with slightly lower odds of depression (OR [95% CI] = 0.97 [0.91; 1.03]; I^2^ = 48%), cardiometabolic disease (OR [95% CI] = 0.86 [0.73; 1.01]; I^2^ = 93%), and their comorbidity (OR [95% CI] = 0.69 [0.66; 0.73]; I^2^ = 1%) in the reference group (no maltreatment), although the effect was only meaningful for comorbidity (Fig. 3, left column).

Physical activity was also linked to lower odds of depression (OR [95% CI] = 0.94 [0.92; 0.96]; I^2^ = 0%), cardiometabolic disease (OR [95% CI] = 0.88 [0.75; 1.04]; I^2^ = 92%), and comorbidity (OR [95% CI] = 0.93 [0.74; 1.17]; I^2^ = 66%; Fig. 2, middle column) in older adults. Effect sizes were overall small and only meaningful for depression. As expected, smoking was a risk factor for all three outcomes (Fig. 2, right column), with largest effects for comorbidity (OR [95% CI] = 1.32 [1.08; 1.63]; I^2^ = 79%), followed by cardiometabolic disease (OR [95% CI] = 1.26 [1.07; 1.47]; I^2^ = 94%) and depression (OR [95% CI] = 1.16 [1.04; 1.31]; I^2^ = 82%).

### Sensitivity analyses

When lifestyle behaviours were dichotomised following recommended guidelines, associations remained similar, but with wider confidence intervals (especially for the interaction effects; Additional file 1: Fig. S2-4) [46–48]. Participants who engaged in recommended levels of physical activity were still less likely to be diagnosed with disease, while smokers continued to be at risk of either one or both disease(s) (Additional file 1: Fig. S3) [46–48]. Effect sizes of these dichotomised lifestyle behaviours were overall stronger and meaningful across more outcomes, compared to analyses where physical activity or smoking were modelled continuously. Problematic alcohol intake showed a near-null association across outcomes. One meaningful interaction between childhood maltreatment and smoking on comorbidity was identified (Additional file 1: Fig. S4) [46–48], indicating a stronger association between maltreatment and comorbidity in smokers compared to non-smokers (OR [95% CI] = 1.29 [1.02; 1.63]). Wider confidence intervals for interaction effects were likely caused by very small subgroup sample sizes. For example, there were few participants, who 1) had a history of maltreatment, 2) problematic alcohol intake, and 3) a co-occurring diagnosis of depression and cardiometabolic disease. With this caveat in mind, childhood maltreatment remained a risk factor for all three outcomes, with the largest effects on comorbidity, regardless of any (potentially moderating) effects of lifestyle behaviours (Additional file 1: Fig. S2) [46–48]. Main effects of childhood maltreatment and lifestyle behaviours also remained robust when excluding the interaction term (Additional file 1: Fig. S5-6).

Results stayed consistent when excluding UK Biobank as the largest contributing cohort (Additional file 1: Fig. S7-9). For example, although the associations between alcohol intake and disease outcomes were often the strongest and most consistent (e.g., the narrowest CIs) in UK Biobank, patterns remained similar without UK Biobank. Overall, even after excluding UK Biobank, 1) no childhood maltreatment by lifestyle behaviour interaction effects were detected for any of the three outcomes (Additional file 1: Fig. S7); 2) alcohol intake and physical activity were associated with slightly lower odds of disease, while smoking was linked to higher odds (Additional file 1: Fig. S8); and 3) childhood maltreatment remained a risk factor for all three outcomes (Additional file 1: Fig. S9).

Analyses in unimputed data showed overall similar patterns, although confidence intervals were very large, likely driven by small subgroup sample sizes. As before, no meaningful interaction effects were detected (Additional file 1: Fig. S10). Lifestyle behaviour main effects remained overall similar in direction, yet only the smoking effect on depression and cardiometabolic disease stayed meaningful (Additional file 1: Fig. S11). Childhood maltreatment remained a meaningful risk factor for depression in older adults (Additional file 1: Fig. S12).

## Discussion

Leveraging four large longitudinal cohorts, we analysed data from 181,423 individuals to establish whether lifestyle behaviours might modify the association of childhood maltreatment with depression, cardiometabolic disease, and their comorbidity. Our study provides novel insights into the moderating role of lifestyle behaviours in these risk associations in older adults, extending our previous research on 1) the role of childhood maltreatment on comorbid depression and cardiometabolic disease, and 2) the absence of the moderating effects of lifestyle behaviours on this association in children [8, 16]. Given the large sample size, we find compelling evidence to show that lifestyle behaviours did not moderate the risk association between childhood maltreatment and comorbid depression and cardiometabolic disease in adulthood. Smokers were 16% to 32% more likely to have (comorbid) depression and cardiometabolic disease, compared to non-smokers. Physical activity was linked to a 6% decrease in the odds of depression, but there was no strong evidence of this association for cardiometabolic disease and comorbidity. Conversely, alcohol intake was associated with being 31% less likely to have comorbid depression and cardiometabolic disease, but there was no strong evidence for the association with depression and cardiometabolic disease alone. Individuals with a history of childhood maltreatment were 31% to 100% more likely to have (comorbid) depression and cardiometabolic disease, compared to those without such a history, consistent with our previous studies [8, 16] and other existing research [8, 13–15].

Despite the discernible main effects of lifestyle behaviours on mental and physical health outcomes, it is striking that none of these lifestyle behaviours attenuated the association between childhood maltreatment and comorbid depression and cardiometabolic disease in adults. This broadly aligns with our recent research in children suggesting that lifestyle behaviours such as sleep, diet and exercise cannot mitigate the adverse effects of early life stress on psycho-physical health [16]. Interestingly, however, a prospective study using the UK Biobank cohort, Zou et al. [50], found that adopting four or more healthy lifestyle behaviours together such as smoking, physical activity, diet, sleep, and two forms of social support (social or leisure activities, and friend or family visits), were associated with at least a 25% decrease in the risk of developing cardiovascular disease in the context of early adversity. The current study provided a more fine-grained evaluation of the moderating effects of each lifestyle behaviour. However, the potential synergistic effects of multiple lifestyle behaviours on the association between childhood maltreatment and comorbid depression and cardiometabolic disease warrants further investigation.

Another important relationship to consider is the possible mediating, rather than moderating, effects of lifestyle behaviours. For instance, smoking has been identified as a mediator in the relationship between childhood maltreatment and cardiovascular disease [51]. To our knowledge, there is currently no investigation into the mediating effects of smoking, physical activity, and alcohol intake in the association between childhood maltreatment and comorbid depression and cardiometabolic disease, highlighting a gap in the literature and a need for future studies.

The associations between physical activity or smoking, and comorbid depression and cardiometabolic disease aligned with expectations. The effects of physical activity supports research showing that replacing 30 min per day of sedentary behaviour with light to moderate to vigorous physical activity can decrease the odds of depression by 13% to 19% [52]. Smoking exhibited harmful effects consistent with longitudinal research indicating that persistent daily smoking and a lifetime history of smoking increases the risk of these conditions by > 24% [53, 54].

Caution is advised when interpreting the associations of alcohol, given the well-replicated links between alcohol and liver cirrhosis, cancer, and coronary artery disease [55]. In our study, alcohol intake was associated with decreased odds of comorbid depression and cardiometabolic disease, a finding that may coincide with reports that the relationship between alcohol and poor physical health outcomes might follow a J-shaped curve, with less harmful effects of moderate alcohol consumption [56–58]. However, we tested only linear associations and several factors should also be considered. For example, the increased risk of depression and cardiometabolic disease is more likely influenced by the type of alcohol consumed [59–61], as well as the amount of intake on a given day of drinking rather than the frequency of consumption [62]. High alcohol intake is also linked to participant attrition, which itself is associated with mortality and heart disease. Thus, our results might be affected by possible survival bias [63]. Further research incorporating these considerations is required to disentangle the complex relationship between alcohol intake and health outcomes.

This study had major strengths. The meta-analysis was based on large cohorts across the UK and Netherlands, making this one of the largest investigations on the associations between childhood maltreatment, lifestyle factors, and health outcomes to date. The inclusion of cohort studies with different designs (population-based and case–control) as well as the consistency of effects across cohorts also highlights the robustness of our findings. Although, the varied designs across cohorts introduced heterogeneity between measures, we addressed this by binarizing lifestyle behaviours in sensitivity analyses using recommended guidelines and found similar results. We excluded, where possible, individuals with pre-existing depression at baseline to mitigate the risk of reverse causation (i.e., engaging in more beneficial lifestyle behaviours after receiving a disease diagnosis). However, the resulting samples may have included healthier, and more resilient individuals who developed comorbid depression and cardiometabolic disease only later in life. Hence, as we specified, our results may be specific to comorbidity in older adults. Furthermore, our findings are based on observational data, limiting our ability to gain insights into potentially causal effects. Future studies should follow up on our findings using stronger casual inference designs, such as randomized controlled trials.

Other limitations include the use of retrospective reports. For instance, retrospective childhood maltreatment measures do not always align with prospective third-party observations of childhood maltreatment, each possibly specific to certain groups and risk pathways [64]. However, first, our findings are in line with Defina et al. [16] which used prospective measures. Second, retrospective reports are often more easily available in datasets covering older adults with disease outcomes relevant to our study. Additionally, although we did covary for educational attainment as a harmonizable proxy measure for socioeconomical status (SES), in line with previous literature suggesting that educational attainment and SES are highly correlated [65, 66], broader measures of SES were not covaried for in this study which may have impacted our findings. For example, evidence suggests that high SES (e.g., education, employment status, and income) is associated with more frequent alcohol intake, but low SES is associated with alcohol-related mortality [67, 68]. Future research incorporating broader measures of SES as a covariate is needed.

## Conclusions

Of the three lifestyle behaviours examined in our sample (smoking, physical activity, and alcohol intake), none moderated the association between childhood maltreatment and comorbid depression and cardiometabolic disease. Smoking was a consistent risk factor for all health outcomes, however, there were mixed effects for alcohol intake and physical activity. While we confirmed that childhood maltreatment was linked to a higher risk of comorbid depression and cardiometabolic disease, following our previous studies by Souama et al. [8] and Defina et al. [16], this had no baring on the risk associations between the lifestyle behaviours measured and the odds of these conditions. These findings underscore the impact of these lifestyle behaviours on mental and physical health regardless of ones history of childhood maltreatment. This also highlights the need for further research aimed at investigating these complex relationships, and at elucidating intervention targets (e.g., diet or social support) that might ameliorate the adverse health outcomes associated with childhood maltreatment.

## Supplementary Information

Additional file 1.

## Data Availability

The code used for statistical analyses in this study is publicly available: https://github.com/stegosaurusrox/childhood.maltr_lifestyle_multimorb_adults. The individual participant data from all cohorts that support the findings of this study are available from management teams of UK Biobank, NESDA, and ALSPAC, but restrictions apply to the availability of these data, which were used under license for the current study, and so are not publicly available. Data are however available from the authors upon reasonable request and with permission of the management teams of UK Biobank, NESDA, and ALSPAC. Additionally, for ALSPAC, please note that the study website contains details of all the data that is available through a fully searchable data dictionary and variable search tool: http://www.bristol.ac.uk/alspac/researchers/our-data/. Derived aggregate data are available upon reasonable request from authors and with permission from each cohort.

## Abbreviations

ALSPAC: Avon Longitudinal Study of Parents And Children
CI: Confidence interval
I^2^: Measure of heterogeneity
ISCED: International Standard Classification of Education
M: Months
MET: Metabolic equivalent total
NESDA: Netherlands Study of Depression and Anxiety
N_pooled_: Pooled sample size
OR: Odds ratio
SES: Socioeconomical status
Y: Years
