# Supplementary material for: Lifestyle behaviours do not moderate the association between childhood maltreatment and comorbid depression and cardiometabolic disease in older adults: a meta-analysis
Source: BMC Med. 2025 Mar 5;23:133. doi: 10.1186/s12916-025-03950-1 (PMC11881505; doi:10.1186/s12916-025-03950-1)
Supplement: Supplementary file 1 — Additional file 1. [file 12916_2025_3950_MOESM1_ESM.docx]

**Additional file 1 for:**

**Lifestyle behaviours as modifiers in the risk association between childhood maltreatment and depressive-cardiometabolic comorbidity in older adults: a meta-analysis**

**Contents**

Section 1 Methods……………………………………………………….……………………………………..........................…… 2

Section 1.1 Study descriptions…………………………………………………………..........................………… 2

Section 1.2 Measures………………………………………………………………………………..........................… 4

Section 2 Results………………………………………………………………………………………………….........................… 15

**Additional file Section 1 Methods**

Section 1.1 Study descriptions (UK Biobank, NESDA, and ALSPAC mothers and partners)

*UK Biobank*

The UK Biobank is a longitudinal cohort study with over 500,000 participants. Participants aged 40-69, were identified from National Health Service records and recruited in 2006-2010 across 22 centres in the UK. At the baseline assessment, participants completed extensive assessments, including pre-existing health conditions, self-report questionnaires, a range of physical measures, and a collection of biological samples.

The UK Biobank study (application number 65769) obtained generic approval from the National Health Service, National Research Ethics Service (17 June 2011, Ref 11/NW/0382). Participants gave full informed written consent and had the right to withdraw at any time.

*Netherlands Study of Depression and Anxiety* (*NESDA)*

NESDA is an ongoing multi-centre cohort study investigating long-term outcomes of depressive and anxiety disorders. 2,981 participants aged 18-65, were recruited from community (n = 807), primary care (n = 564), and specialised mental health settings (n = 1610). Participants consisted of healthy controls and individuals with a current or past depressive or anxiety disorder. At baseline, participants were assessed on the presence, severity, categorisation, and symptoms of their depressive or anxiety disorder, as well as various factors deemed important determinants of mental health outcomes such as lifestyle behaviours. Follow up assessments were conducted biannually.

The protocol of the Netherlands Study of Depression and Anxiety was approved centrally by the Ethical Review Board of the VU University Medical Centre and subsequently by local review boards of each participating center. All participants provided written informed consent.

*Avon Longitudinal Study of Parents And Children (ALSPAC) mothers and partners*

Pregnant women resident in Avon, UK with expected dates of delivery 1st April 1991 to 31st December 1992 were invited to take part in the study. The initial number of pregnancies enrolled was 14,541. Of these initial pregnancies, there were 14,062 live births and 13,988 children who were alive at 1 year of age. When the oldest children were approximately 7 years of age, an attempt was made to bolster the initial sample with eligible cases who had failed to join the study originally. The phases of enrolment are described in more detail in the cohort profile paper and its update. The total sample size for analyses using any data collected after the age of seven is therefore 15,447 pregnancies, resulting in 15,658 fetuses. Of these 14,901 were alive at 1 year of age. Of the original 14,541 initial pregnancies, 338 were from women who had already enrolled with a previous pregnancy, meaning 14,203 unique mothers were initially enrolled in the study. As a result of the additional phases of recruitment, a further 630 women who did not enrol originally have provided data since their child was 7 years of age. This provides a total of 14,833 unique women (G0 mothers) enrolled in ALSPAC as of September 2021. G0 partners were invited to complete questionnaires by the mothers at the start of the study and they were not formally enrolled at that time (data collection for questionnaires was conducted with REDCap. 12,113 G0 partners have been in contact with the study by providing data and/or formally enrolling when this started in 2010. 3,807 G0 partners are currently enrolled.

Ethical approval for the study was obtained from the ALSPAC Ethics and Law Committee and the Local Research Ethics Committees. Informed consent for the use of data collected via questionnaires and clinics was obtained from participants following the recommendations of the ALSPAC Ethics and Law Committee at the time.

Please note that the study website contains details of all the data that is available through a fully searchable data dictionary and variable search tool: <http://www.bristol.ac.uk/alspac/researchers/our-data/>

**Section 1.2 Measures by study**

A more detailed description of exposure (childhood maltreatment) and outcome measures (depression, cardiometabolic disease) can be found elsewhere (see Souama et al.).

*Exposure: Childhood maltreatment*

| Study | Instrument | Response type | Mean age at interview | Period reported on | Definition |  |  |
| --- | --- | --- | --- | --- | --- | --- | --- |
|  |  |  |  |  | Physical abuse (SR) | Emotional abuse (SR) | Sexual abuse (SR) |
| ALSPAC, mothers | Single item | D | 29 years | ‘before you were 17’ | Yes | Yes | Yes |
| ALSPAC, partners | Single item | D | 31 years | ‘before you were 17’ | Yes | Yes | Yes |
| NESDA | CTI | LSF | 42 years | ‘first 16 years of life’ | At least regularly | At least regularly | At least once |
| UKB Biobank | Childhood Trauma Screener | LSF | 57 years | ‘when you were growing up’ | At least regularly | At least regularly | At least once |

## Table S1. *Childhood maltreatment assessment overview.*

*Abbreviations*. CTI: Childhood Trauma Interview. D: dichotomous. LSF: Likert-type scale of frequency. SR: self-reported.

*Outcome: Depression*

| Study | Instrument | Response type | Mean age at interview | Period reported on |
| --- | --- | --- | --- | --- |
| ALSPAC, mothers | EPDS | SR depressive symptomatology scale (Cut-off ≥ 13) | 48 years (= 18 year follow-up after baseline), not yet present at 5 earlier time points which overlapped with the same time period when reports of lifestyle behaviours were made [18 and 32 weeks of pregnancy as well as 8 week, 8 & 21 month follow-ups] | ‘in the past week’ |
| ALSPAC, partners | EPDS | SR depressive symptomatology scale ( Cut-off ≥ 13) | 53 years (=21/22 year follow-up after baseline), not yet present at 5 earlier time points which overlapped with the same time period when reports of lifestyle behaviours were made [18 and 32 weeks of partner’s pregnancy as well as 8 week, 8 & 21 month follow-up] | ‘in the past week’ |
| NESDA | CIDI, version 2.1 | Clinical interview | 44-48 years (2, 4 or 6 year follow-up), not yet present at baseline when reports of lifestyle behaviours were made (42y) | ‘ever’ |
| UKBB | Two items (‘seen a doctor / psychiatrist’) | D, SR | 61-66 years (6, 8 or 11 year follow-up), not yet present at baseline when reports of lifestyle behaviours were made (57 years) | ‘ever’ (after baseline) |

Table S2. Depression assessment overview.

*Abbreviations*. CIDI: composite international diagnostic interview D: dichotomous. EPDS: Edinburgh postnatal depression scale SR: self-reported.

##

*Outcome: Cardiometabolic disease*

| Study | Instrument | Response type | Mean age at interview | Period reported on |
| --- | --- | --- | --- | --- |
| ALSPAC, mothers | Three items (heart attack, stroke, diabetes) | D, SR | 48 years | ‘ever’ |
| ALSPAC, partners | Three items (heart attack, stroke, diabetes) | D, SR | 53 years | ‘ever’ |
| NESDA | Three items (heart condition or heart infarct, stroke or cerebral hemorrhage, or diabetes) | D, SR | 44-48 (2, 4 or 6 year follow-up), not yet present at baseline when reports of lifestyle behaviours were made (42 years) | ‘ever’ |
| UKBB | Four items (diabetes, heart attack, angina, stroke, blood clot) | D, SR | 61-66 (6, 8 or 11 year follow-up), not yet present at baseline when reports of lifestyle behaviours were made | ‘ever’ (after baseline) |

Table S3. Cardiometabolic disease assessment overview.

*Abbreviations.* D: dichotomous. SR: self-reported.

*Moderators: Lifestyle behaviours*

*Smoking*

| Study | Instrument | Response type | Mean age at interview | Period reported on |
| --- | --- | --- | --- | --- |
| ALSPAC, mothers | Single item (number of cigarettes smoked per day) | C, SR | Averaged across 4 time points (21, 33, 61 and 85 month follow-ups) | Currently, per day |
| ALSPAC, partners | Single item (number of cigarettes smoked per day) | C, SR | Averaged across 4 time points (8, 21, 33 and 61 month follow-ups) | Currently, per day |
| NESDA | Single item (number of cigarettes smoked per day) | C, SR | 42 years | Currently, per day |
| UKBB | Single item (number of cigarettes smoked per day) | C, SR | 57 years | Currently, per day |

Table S4. Smoking assessment overview.

*Abbreviations.* C: continuous. SR: self-reported

*Alcohol intake*

| Study | Instrument | Level coding | Response type | Mean age at interview | Period reported on |
| --- | --- | --- | --- | --- | --- |
| ALSPAC, mothers | Single item (‘how much alcohol do you drink?’) | 1 = Never drink alcohol  2 = Very occasionally (less than once a week)  3 = Occasionally (at least once a week)  4 = Drink 1-2 glasses nearly every day  5 = Drink 3-9 glasses every day  6 = Drink at least 10 glasses a day | LSF, SR | Averaged across 3 time points (21, 33, and 61 month follow-ups) | Currently, per day |
| ALSPAC, partners | Single item (‘how much alcohol do you drink?’) | 1 = Never drink alcohol  2 = Very occasionally (less than once a week)  3 = Occasionally (at least once a week)  4 = Drink 1-2 glasses nearly every day  5 = Drink 3-9 glasses every day  6 = Drink at least 10 glasses a day | LSF, SR | Averaged across 4 time points (21, 33, 47 and 61 month follow-up) | Current |
| NESDA | Single item (‘how often do you have a drink containing alcohol?’) | 1 = Never  2 = Monthly or less  3 = Two to four times per month  4 = Two to three times per week  5 = Four or more times per week | LSF, SR | 42 years | Current |
| UKBB | Single item (‘about how often do you drink alcohol?’) | 0 = Never  1 = Special occasions only  1 = One to three times a month  2 = Once or twice a week  3 = Three or four times a week  4 = Daily or almost daily | LSR, SR | 57 years | Current |

Table S5. Alcohol intake assessment overview.

*Abbreviations*. C = continuous. LSF: Likert-type scale of frequency. SR: self-reported

*Physical activity*

| Study | Instrument | Level Coding | Response type | Mean age at interview | Period reported on |
| --- | --- | --- | --- | --- | --- |
| ALSPAC, mothers | Several items (‘Frequency / amount of time spent swimming/running/cycling/playing tennis/badminton/aerobics/netball/volleyball/basketball/football/hockey/squash’) | Timepoint 1:  1 = Never  2 = Once a month or less  3 = Once a week or less  4 = 2-3 times a week  5 = 4-5 times a week  6 = Most days | LSF, SR | Averaged across 3 time points (7,8 and 11 year follow-ups) | Past week / past year |
|  |  | Time point 2:  1 = >6 hours  2 = 2 - 6 hours  3 = <2 hours  4 = None |  |  |  |
|  |  | Timepoint 3:  1 = Every day  2 = 3-6 times a week  3 = Once or twice a week  4 = 1-3 times a month  5 = Less than once a month  6 = None |  |  |  |
| ALSPAC, partners | Eight items (‘Frequency spent hiking/walking/jogging/running/cycling/aerobics/tennis/squash/badminton/swimming/other energetic leisure activity?’) | 1 = Every day  2 = 3-6 times a week  3 = Once or twice a week  4 = 1-3 times a month  5 = Less than once a month  6 = None | LSF, SR | 7 year follow-up | Past year |
| NESDA | Six items (‘frequency (number of days/week) doing vigorous/moderate physical activity/walking?’ and ‘time spent doing vigorous/moderate physical activity/walking on these days?’) | Number of days per week and number of hours/minutes per day | C, SR | 42 years | Last 7 days |
| UKBB | Three items (‘number of days / hours per time spent doing physical activity’, ‘at or above moderate/vigorous/walking recommendation’; (8) | Average hours per day multiplied by number of days per week doing physical activity | C, D, SR | 57 years | Current |

Table S6. Physical activity assessment overview.

*Abbreviations.* C = continuous. LSF: Likert-type scale of frequency. SR: self-reported

**Section 2 Results**

Fig. S1 **Main effect of childhood maltreatment on depression, cardiometabolic disease, and comorbidity (continuous lifestyle behaviours).**


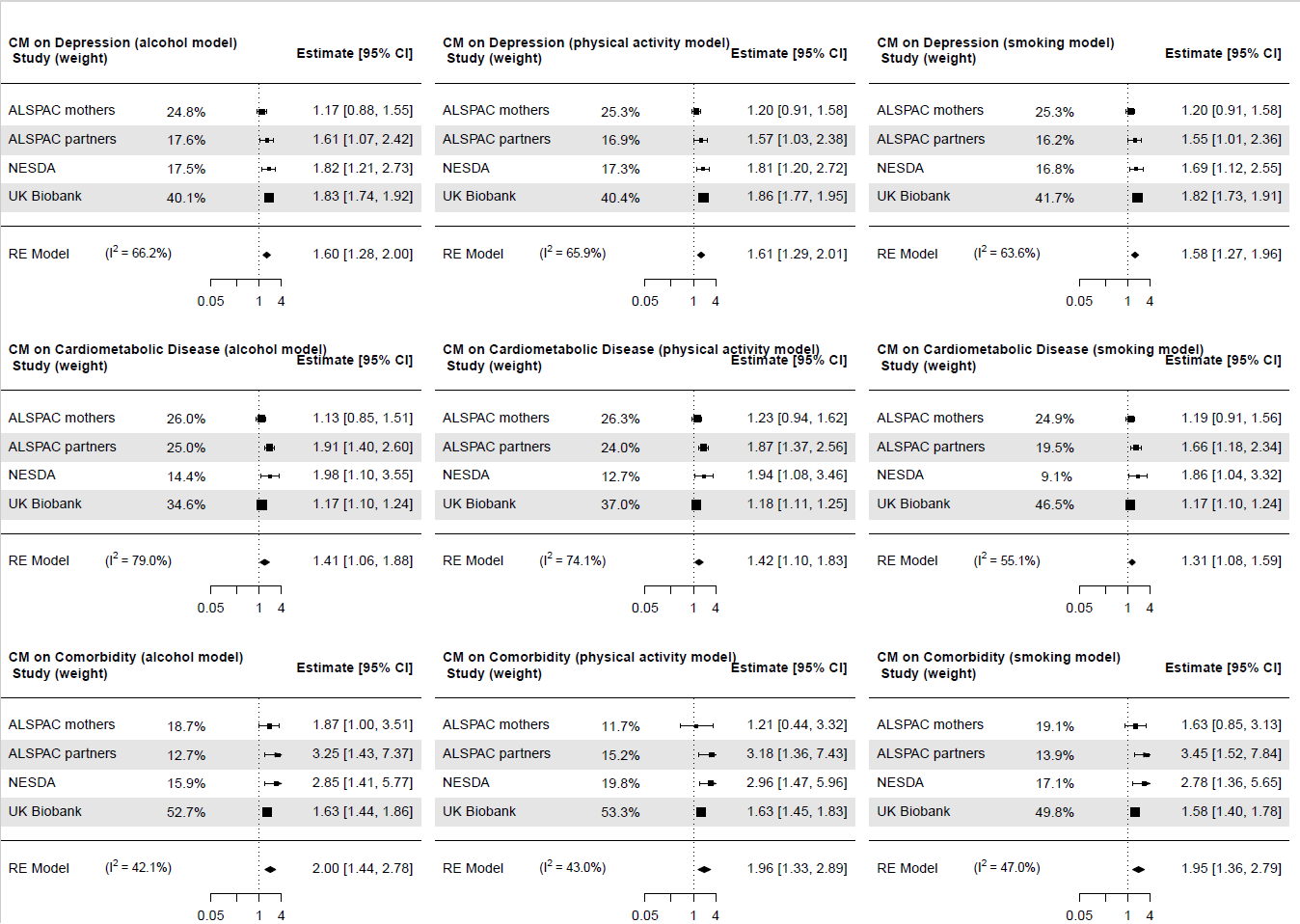


*Note.* Childhood maltreatment main effect on depression (top row), cardiometabolic disease (middle row) and comorbidity (bottom row), accounting for the direct effect of each lifestyle behaviour (alcohol in the left column, physical activity in the middle column, and smoking in the right column) on each outcome. Lifestyle behaviours were modelled continuously.

Fig. S2 **Main effect of childhood maltreatment on depression, cardiometabolic disease, and comorbidity (dichotomous lifestyle behaviours).**


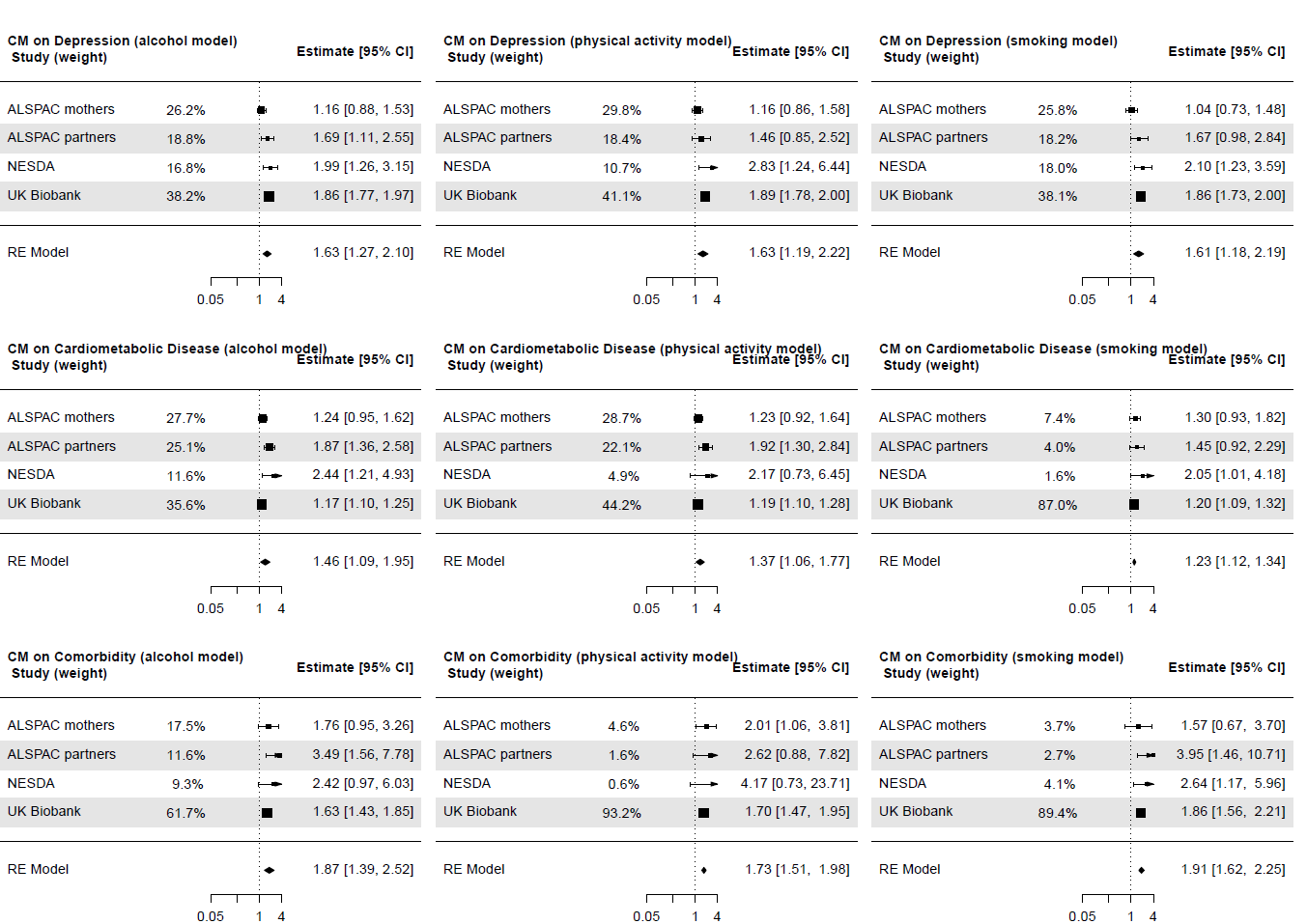


*Note.* Childhood maltreatment main effect on depression (top row), cardiometabolic disease (middle row) and comorbidity (bottom row), accounting for the direct effect of each lifestyle behaviour (alcohol in the left column, physical activity in the middle column, and smoking in the right column) on each outcome. Lifestyle behaviours were dichotomized, following recommended guidelines.

Fig. S3 **Main effect of lifestyle behaviours on depression, cardiometabolic disease, and comorbidity (dichotomous lifestyle behaviours).**


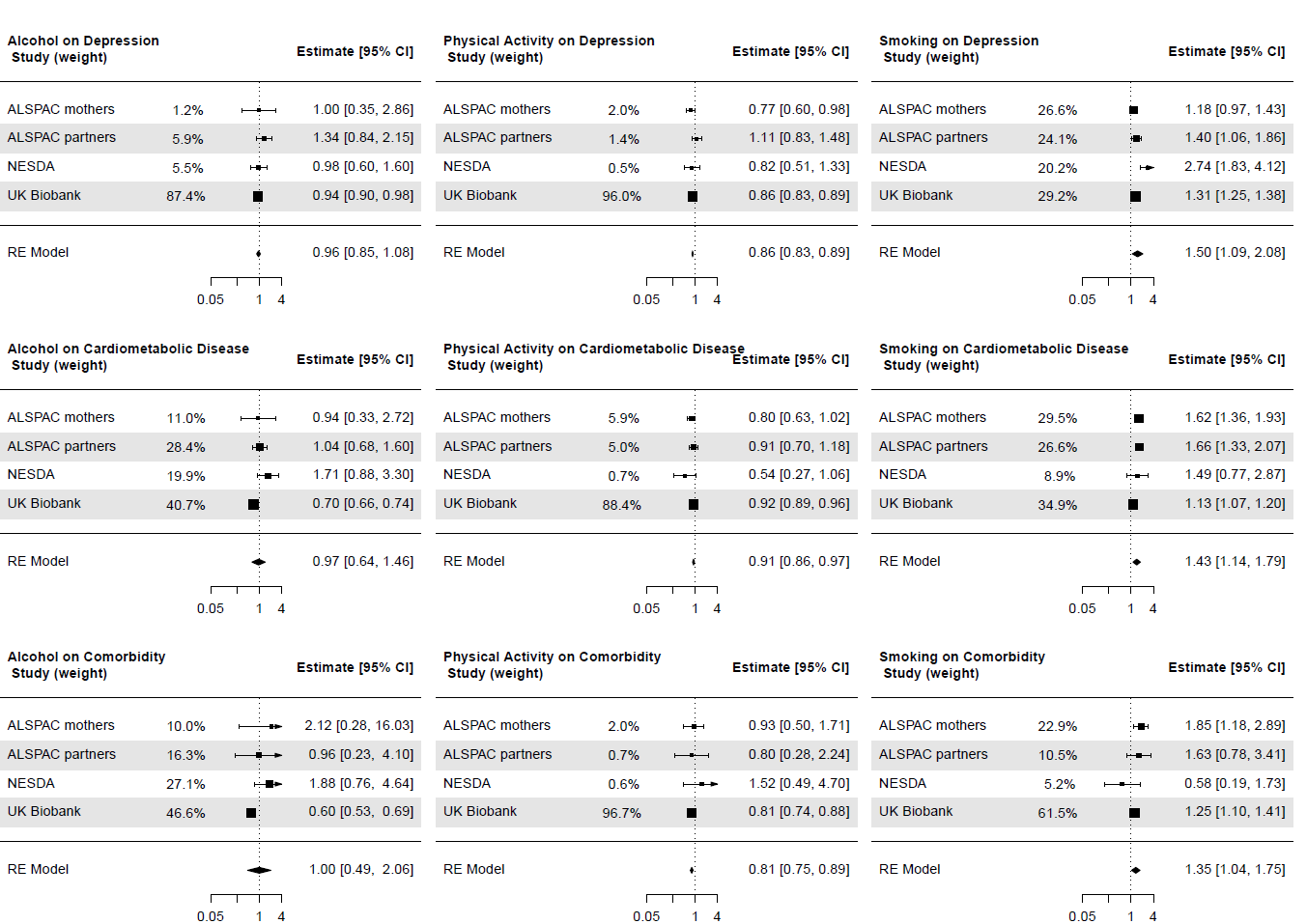


*Note.* Main effect of lifestyle behaviours [alcohol intake (left column), physical activity (middle column) and smoking (right column)] on depression (top row), cardiometabolic disease (middle row) and comorbidity (bottom row). Lifestyle behaviours were dichotomized, following recommended guidelines.

Fig. S4 **Interaction effect of childhood maltreatment by lifestyle behaviours on depression, cardiometabolic disease, and comorbidity (dichotomous lifestyle behaviours).**


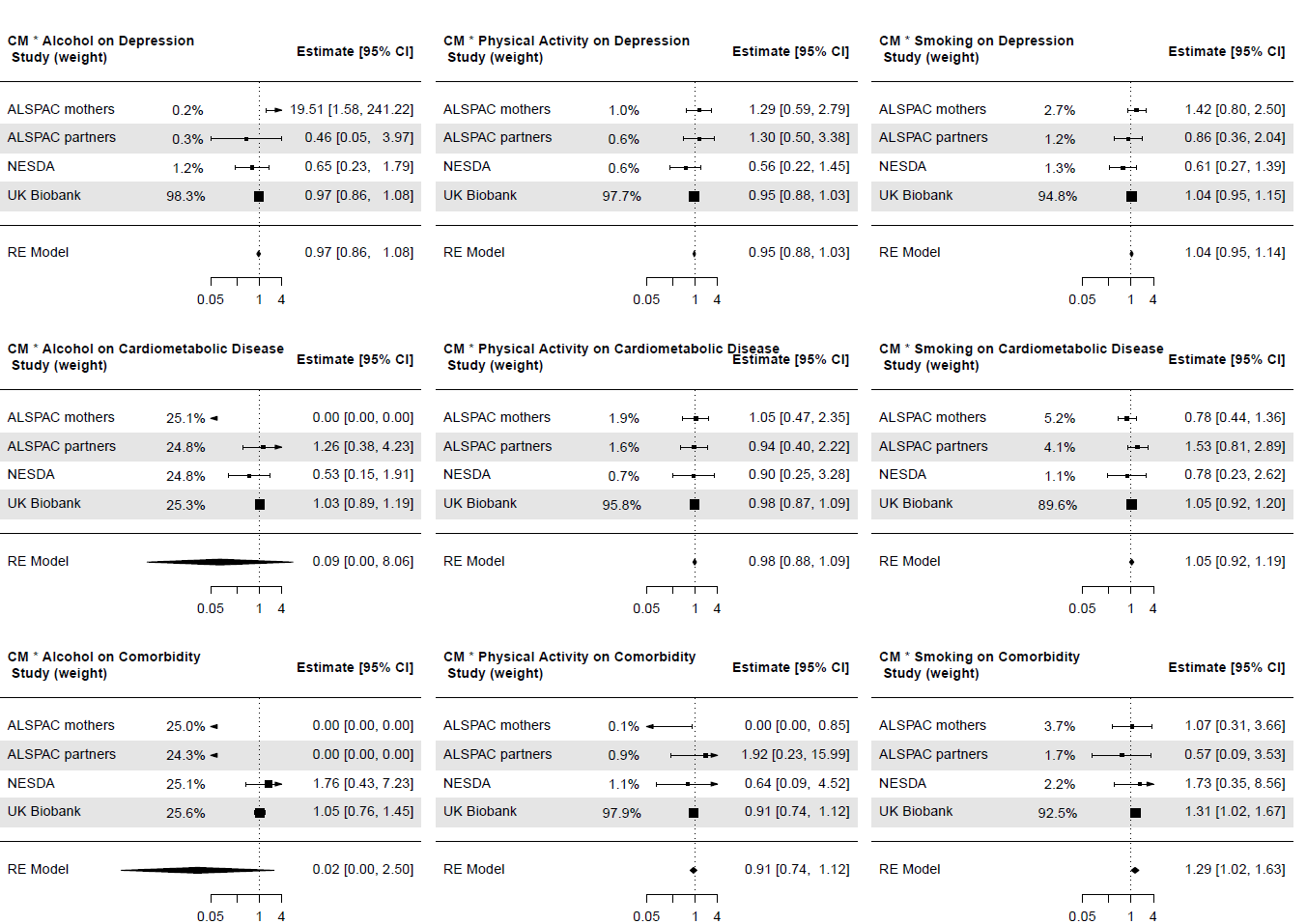


*Note.* Childhood maltreatment by lifestyle behaviour interaction effect on depression (top row), cardiometabolic disease (middle row) and comorbidity (bottom row). Alcohol model shown in the left column, physical activity in the middle column, and smoking in the right column. Lifestyle behaviours were dichotomized, following recommended guidelines.

Fig. S5 **Main effect of childhood maltreatment on depression, cardiometabolic disease, and comorbidity (continuous lifestyle behaviours).**


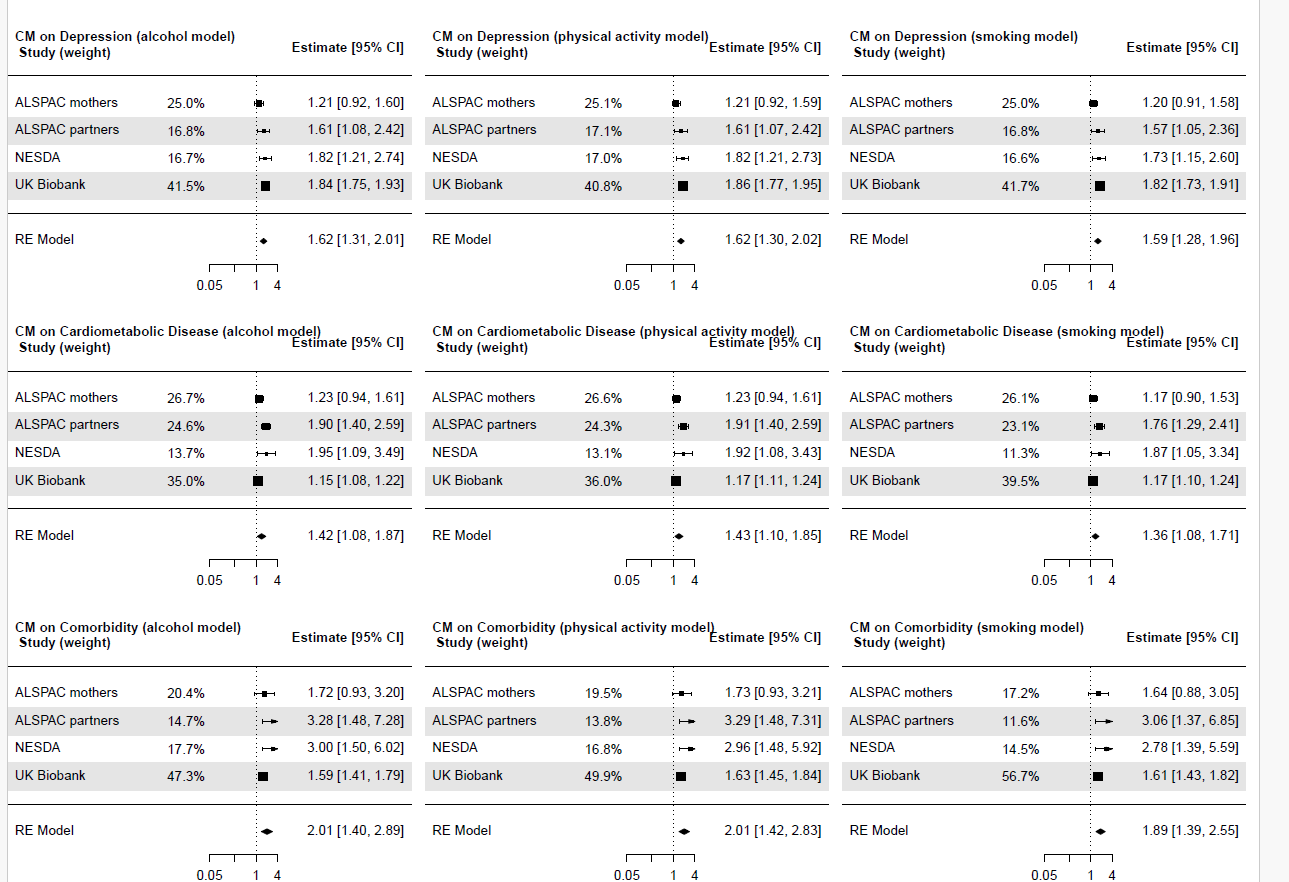


*Note.* Main effect of childhood maltreatment (without including the interaction term) on depression (top row), cardiometabolic disease (middle row) and comorbidity (bottom row). Alcohol model shown in the left column, physical activity in the middle column, and smoking in the right column. Lifestyle behaviours were modelled continuously.

Fig. S6 **Main effect of lifestyle behaviours on depression, cardiometabolic disease, and comorbidity (continuous lifestyle behaviours).**


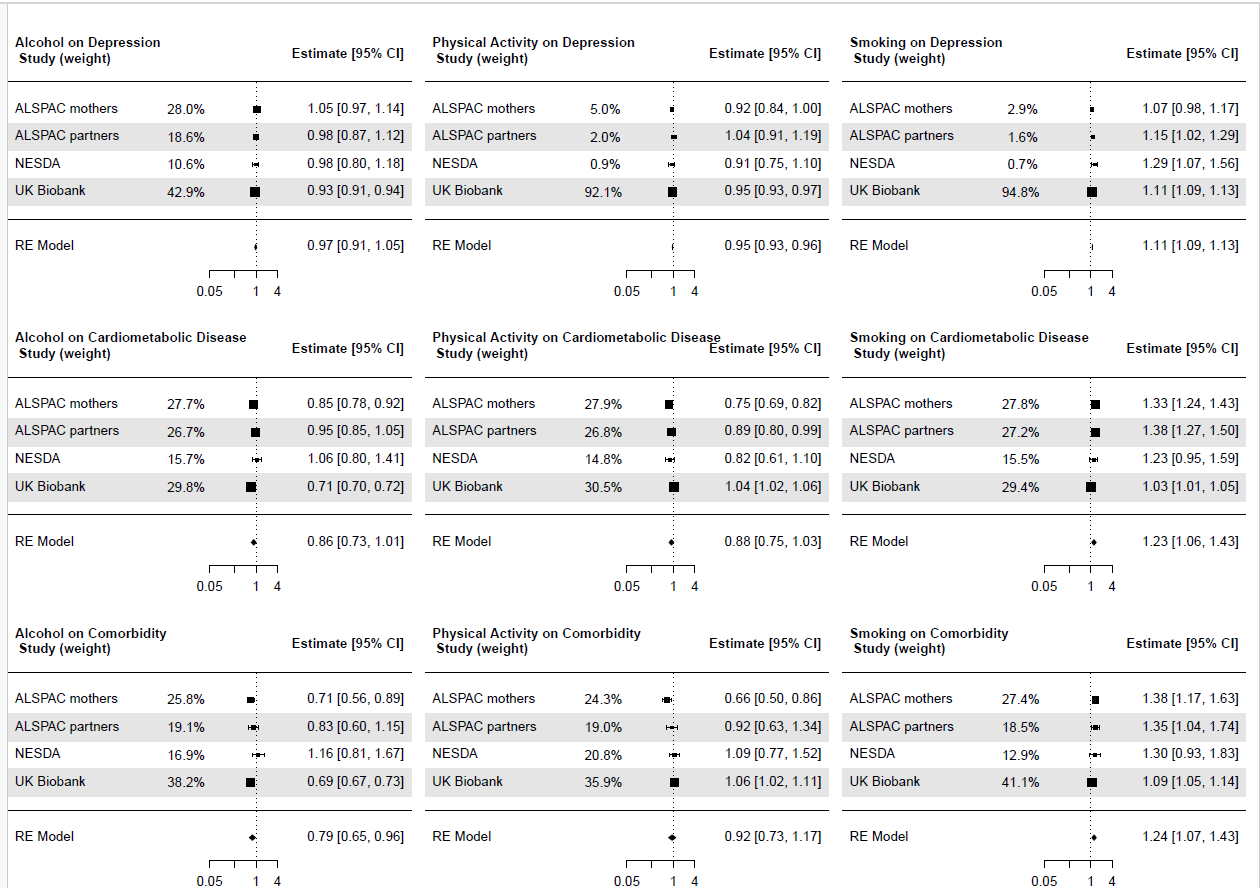


*Note.* Main effect of lifestyle behaviours (without including the interaction term) [alcohol intake (left column), physical activity (middle column) and smoking (right column)] on depression (top row), cardiometabolic disease (middle row) and comorbidity (bottom row). Lifestyle behaviours were modelled continuously.

Fig. S7 **Interaction effect of childhood maltreatment by lifestyle behaviours on depression, cardiometabolic disease, and comorbidity, without UK Biobank (continuous lifestyle behaviours).**


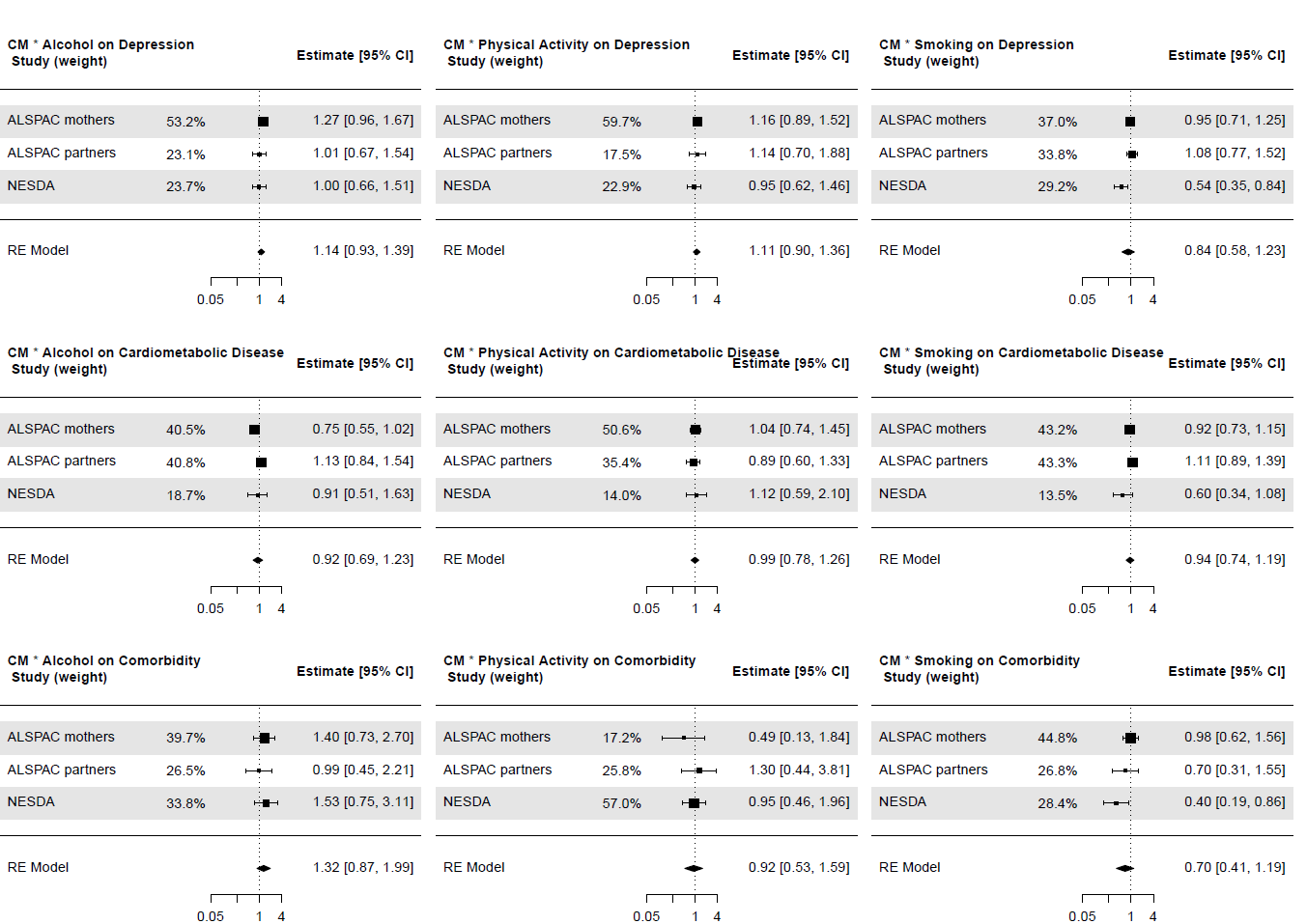


*Note.* Childhood maltreatment by lifestyle behaviour interaction effect on depression (top row), cardiometabolic disease (middle row) and comorbidity (bottom row). Alcohol model shown in the left column, physical activity in the middle column, and smoking in the right column. Lifestyle behaviours were modelled continuously and without UK Biobank.

Fig. S8 **Main effect of lifestyle behaviours on depression, cardiometabolic disease, and comorbidity, without UK Biobank (continuous lifestyle behaviours).**


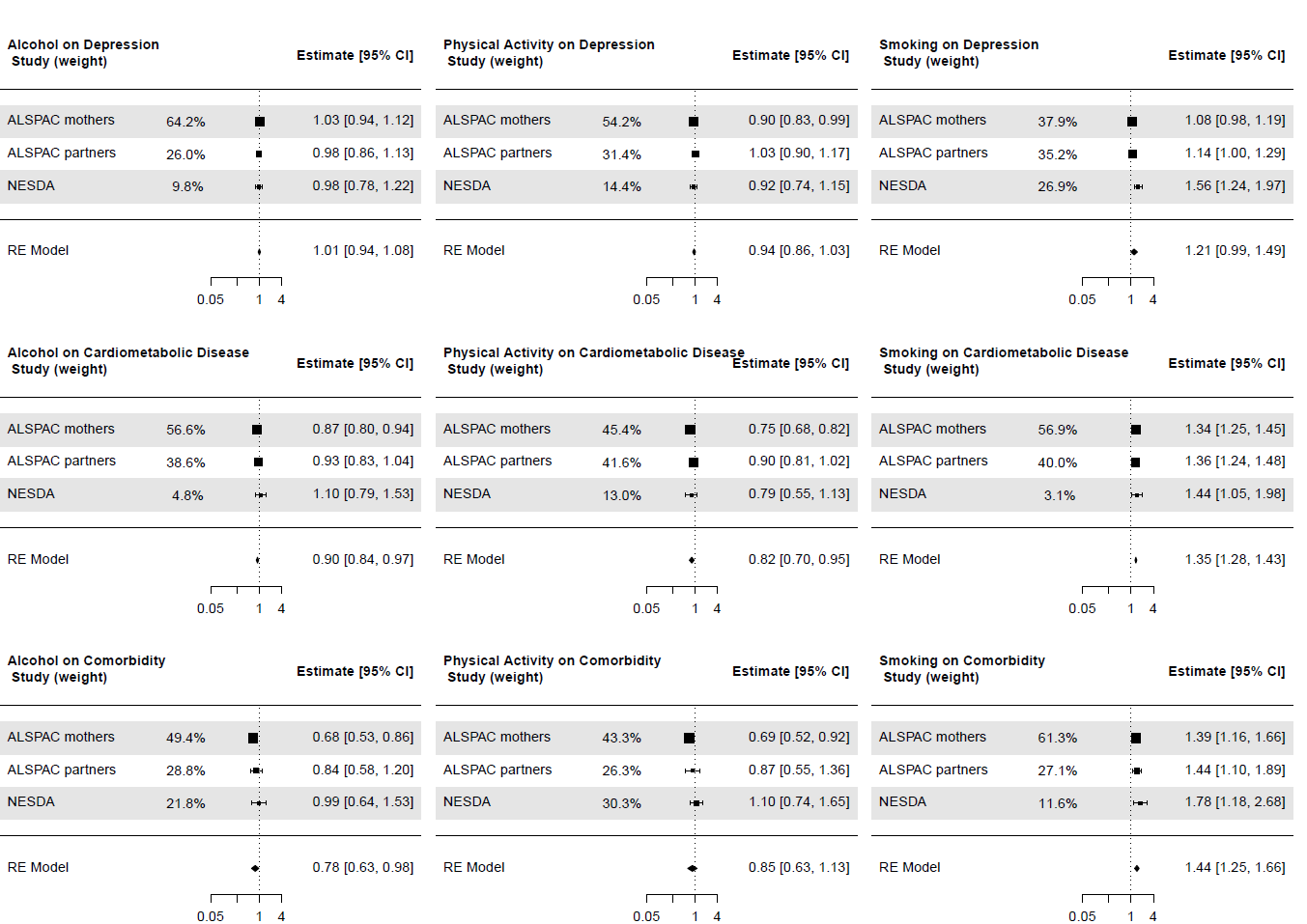


*Note.* Main effect of lifestyle behaviours [alcohol intake (left column), physical activity (middle column) and smoking (right column)] on depression (top row), cardiometabolic disease (middle row) and comorbidity (bottom row). Lifestyle behaviours were modelled continuously and without UK Biobank.

Fig. S9 **Main effect of childhood maltreatment on depression, cardiometabolic disease, and comorbidity, without UK Biobank (continuous lifestyle behaviours).**


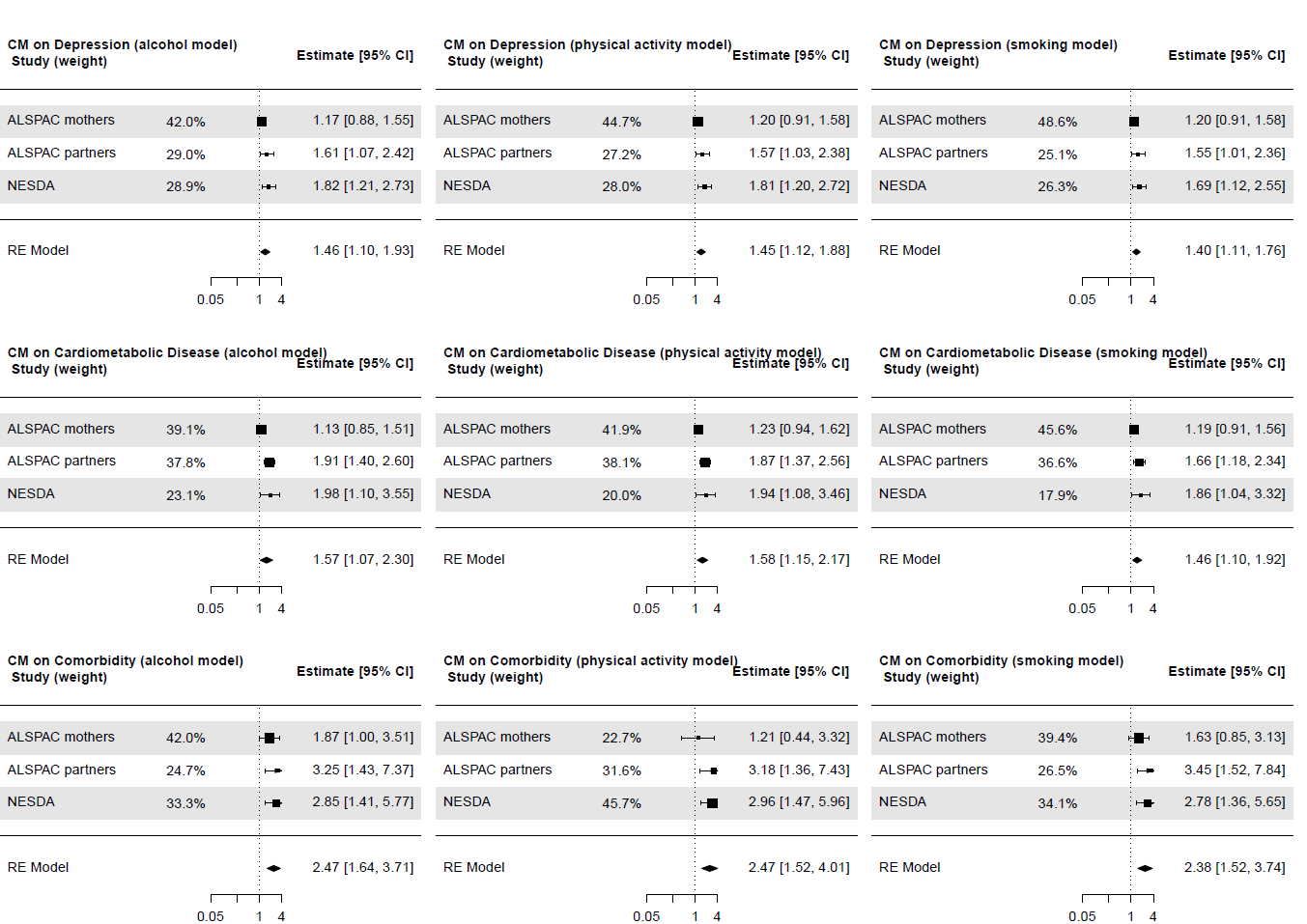


*Note.* Childhood maltreatment main effect on depression (top row), cardiometabolic disease (middle row) and comorbidity (bottom row), accounting for the direct effect of each lifestyle behaviour (alcohol in the left column, physical activity in the middle column, and smoking in the right column) on each outcome. Lifestyle behaviours were modelled continuously and without UK Biobank.

Fig. S10 **Interaction effect of childhood maltreatment by lifestyle behaviours on depression, cardiometabolic disease, and comorbidity using unimputed data (continuous lifestyle behaviours).**
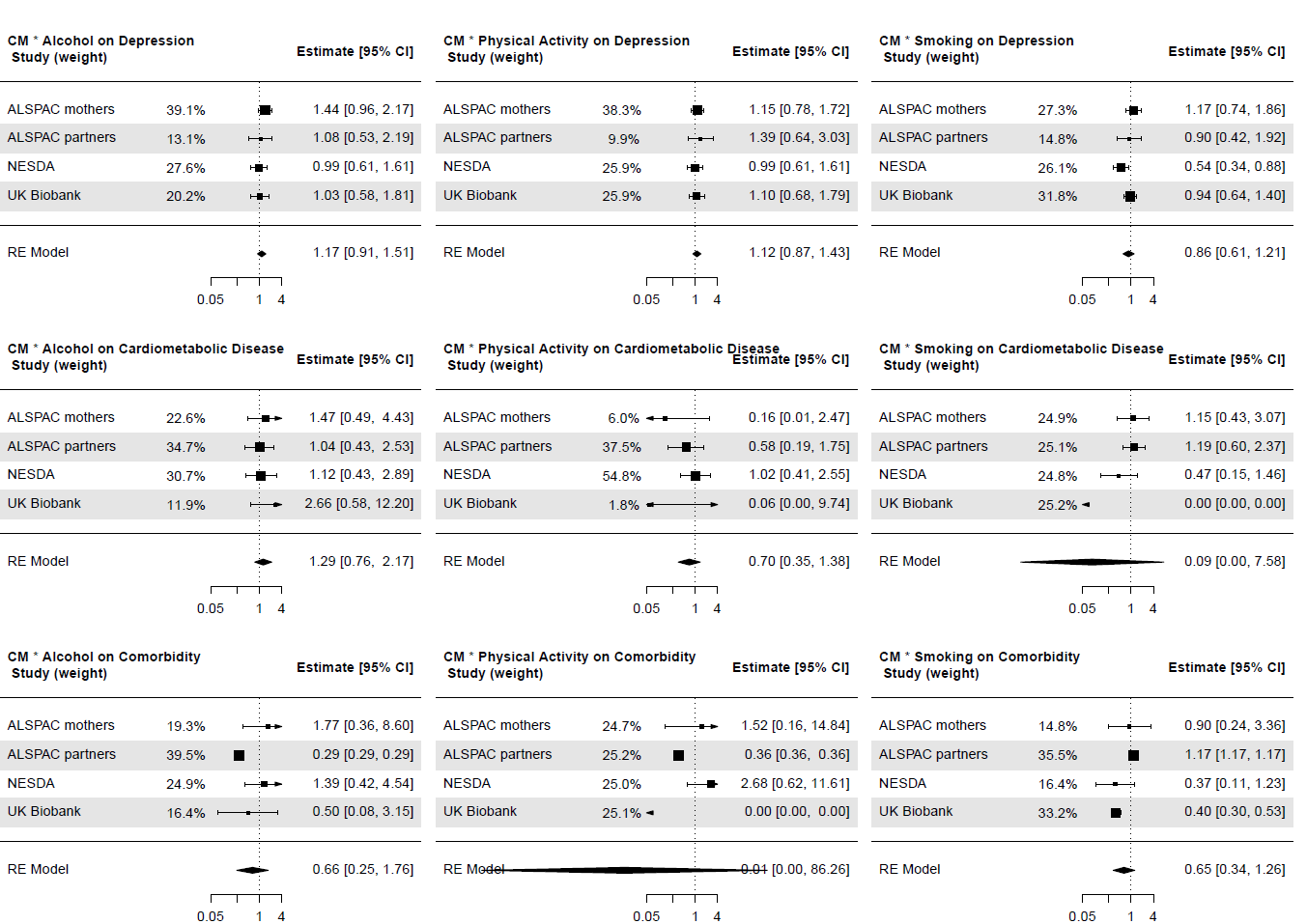


*Note.* Childhood maltreatment by lifestyle behaviour interaction effect on depression (top row), cardiometabolic disease (middle row) and comorbidity (bottom row). Alcohol model shown in the left column, physical activity in the middle column, and smoking in the right column. Lifestyle behaviours were modelled continuously in unimputed data.

Fig. S11 **Main effect of lifestyle behaviours on depression, cardiometabolic disease, and comorbidity, using unimputed data (continuous lifestyle behaviours).**


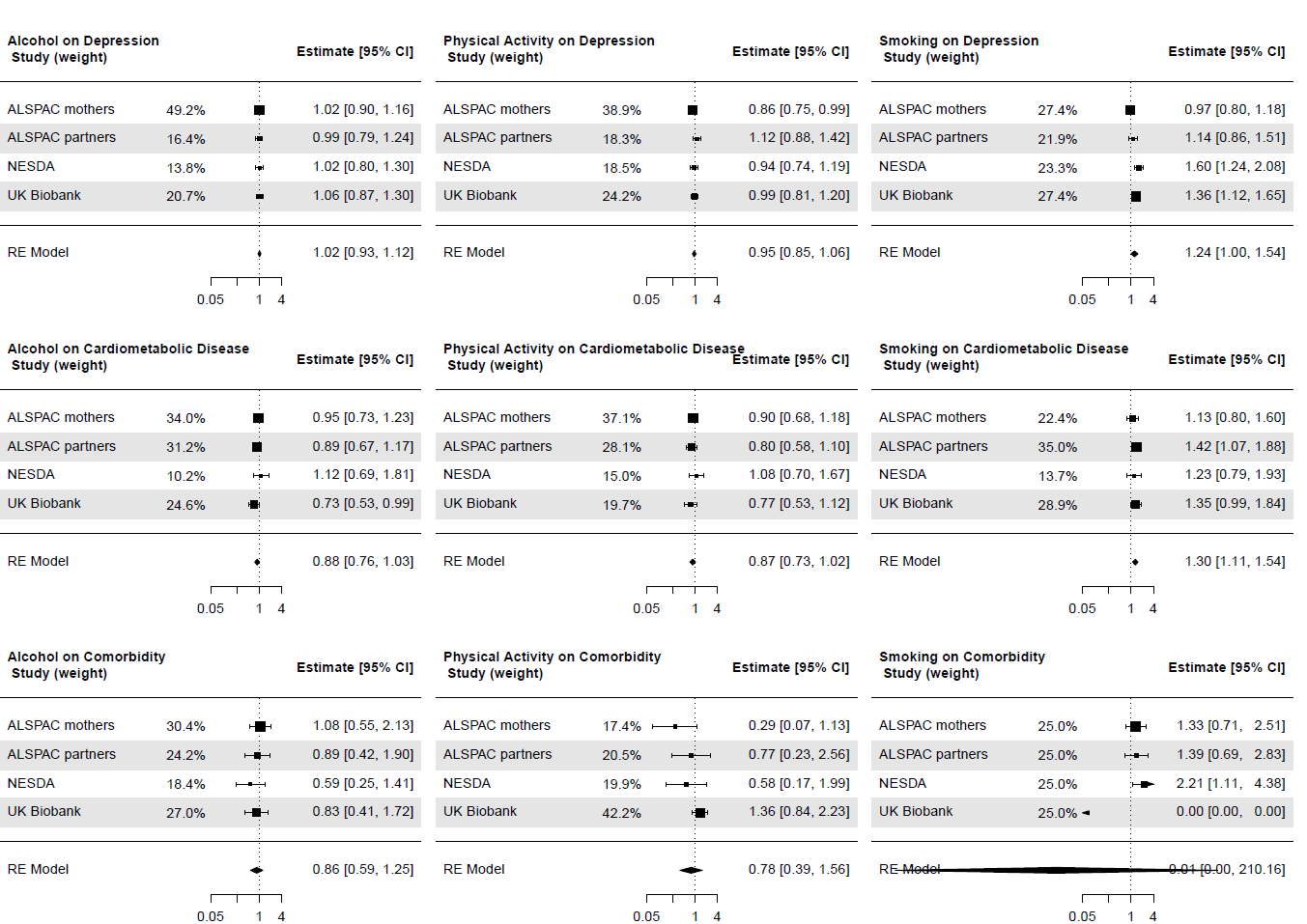


*Note.* Main effect of lifestyle behaviours [alcohol intake (left column), physical activity (middle column) and smoking (right column)] on depression (top row), cardiometabolic disease (middle row) and comorbidity (bottom row). Lifestyle behaviours were modelled continuously in unimputed data.

Fig. S12 **Main effect of childhood maltreatment on depression, cardiometabolic disease, and comorbidity, using unimputed data (continuous lifestyle behaviours).**


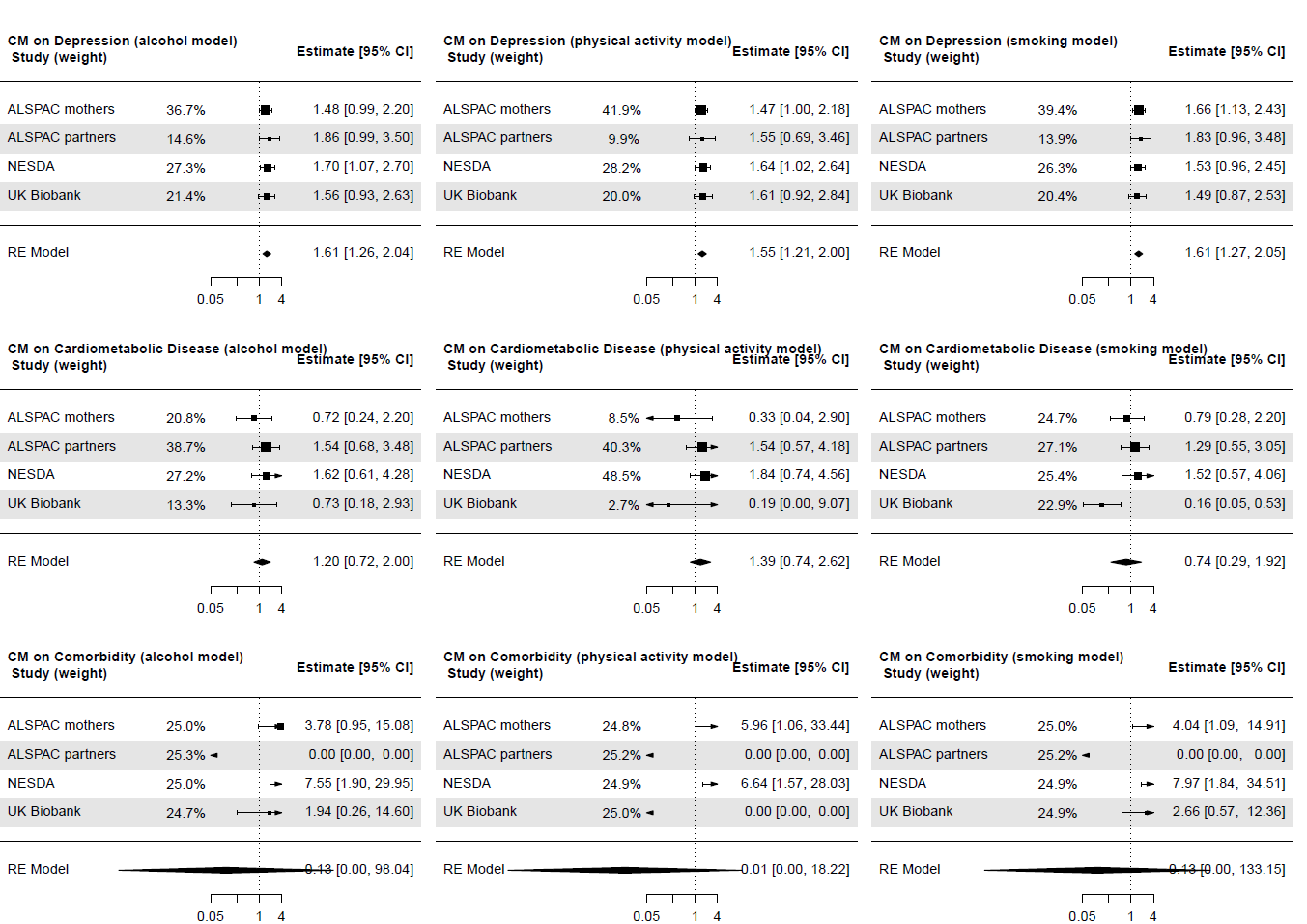


*Note.* Childhood maltreatment main effect on depression (top row), cardiometabolic disease (middle row) and comorbidity (bottom row), accounting for the direct effect of each lifestyle behaviour (alcohol in the left column, physical activity in the middle column, and smoking in the right column) on each outcome. Lifestyle behaviours were modelled continuously in unimputed data.
